# Supplementary material for: Regulating adsorption performance of zeolites by pre-activation in electric fields
Source: Nat Commun. 2023 Sep 6;14:5479. doi: 10.1038/s41467-023-41227-4 (PMC10482906; doi:10.1038/s41467-023-41227-4)
Supplement: Supplementary file 1 — Supplementary Information [file 41467_2023_41227_MOESM1_ESM.pdf]

Supplementary Information

Regulating Adsorption Performance of Zeolites by Pre-activation in Electric Fields

Kaifei Chen et al.

Supplementary Figures

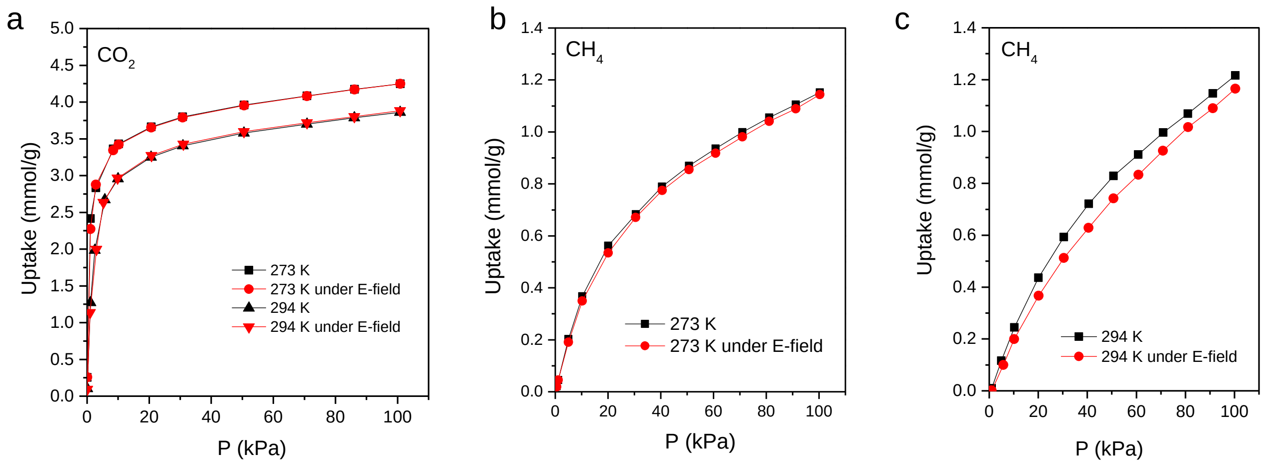

Supplementary Figure 1 Adsorption isotherms of (a) CO_2 and (b-c) CH_4 at 273 K and 294 K in r2KCHA without or with the *in situ* application of a 240 V/mm E-field.

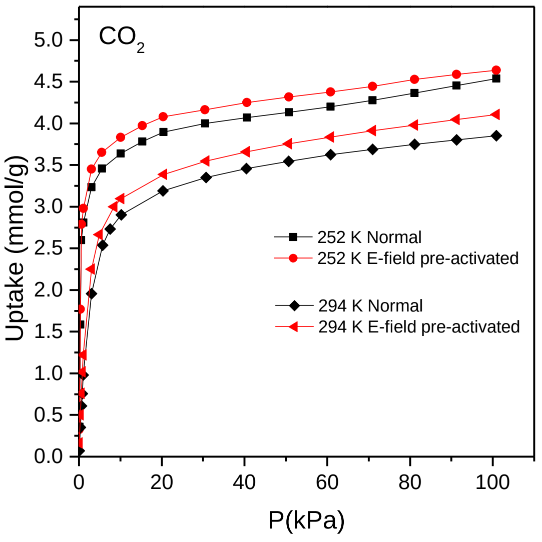

Supplementary Figure 2 Adsorption isotherms of CO_2 in r2KCHA at 252 K and 294 K without and with the E-field pre-activation.

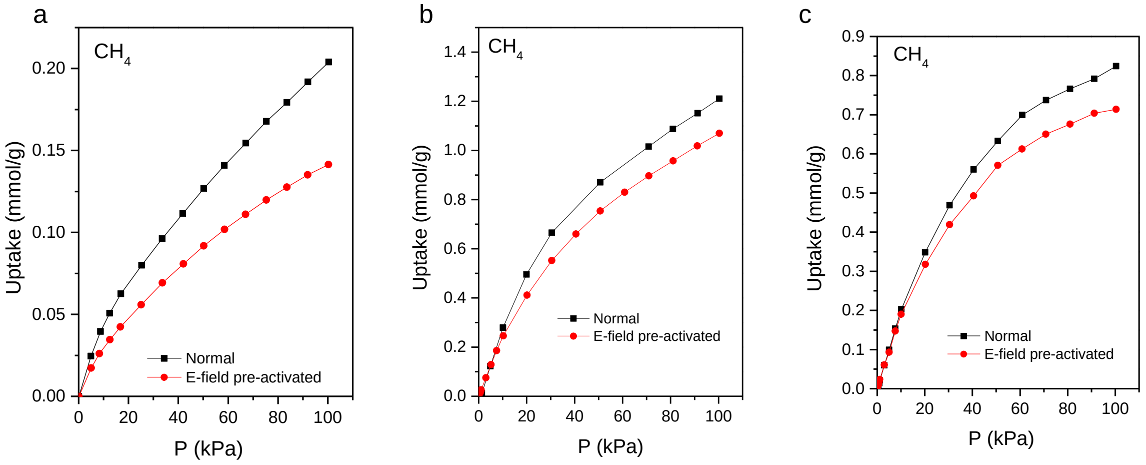

Supplementary Figure 3 Adsorption isotherms of CH_4 in r2KCHA at (a) 252 K, (b) 294 K and (c) 323 K without and with the E-field pre-activation. Because of the trapdoor effect, the uptakes of CH_4 at 273 and 294 K were close, leading to a crossover of the isotherms.

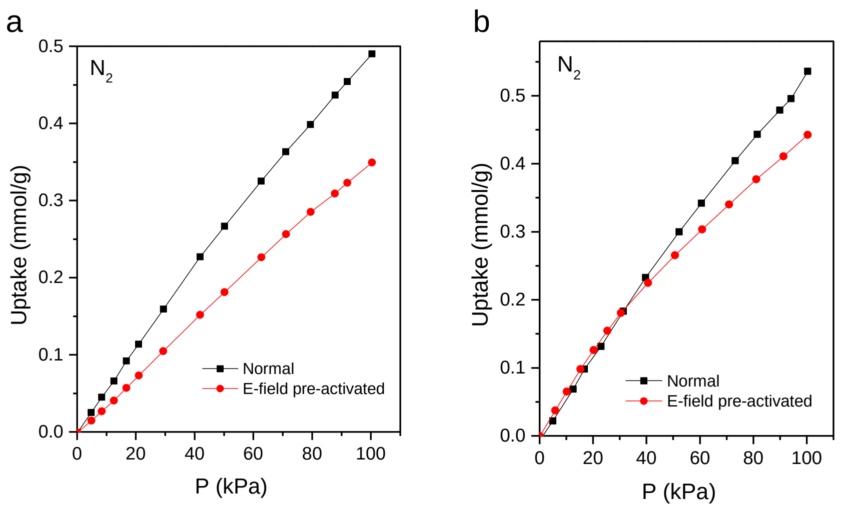

Supplementary Figure 4 Adsorption isotherms of N_2 in r2KCHA at (a) 252 K and (b) 294 K without and with the E-field pre-activation.

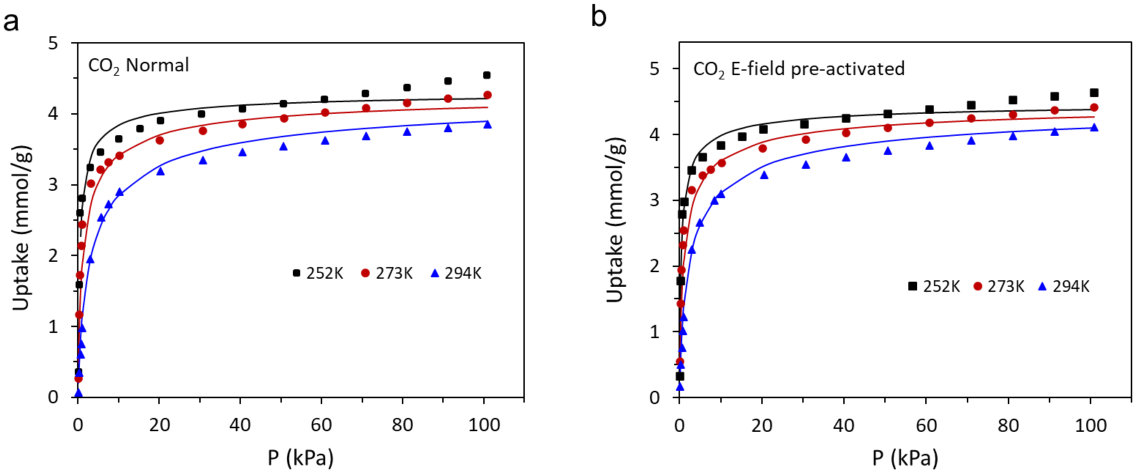

Supplementary Figure 5 Adsorption isotherms of CO_2 in r2KCHA fitted with Toth model (a) without and (b) with the E-field pre-activation. Solid symbols refer to the experimental data, with curves depicting the theoretical-fitting results using the Toth model.

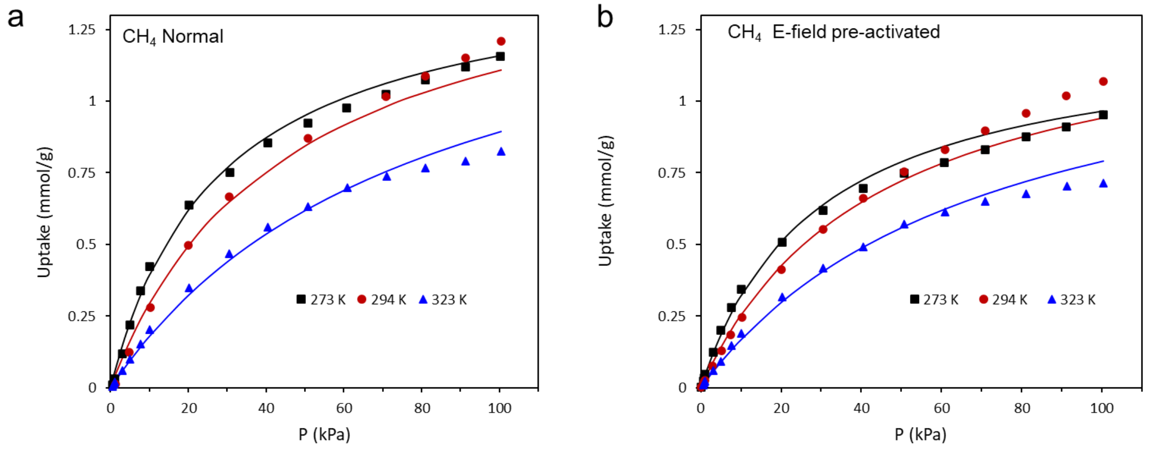

Supplementary Figure 6 Adsorption isotherms of CH_4 in r2KCHA fitted with LJM-Toth model (a) without and (b) with the E-field pre-activation. Solid symbols refer to the experimental data, with curves depicting the theoretical-fitting results using the LJM-Toth model.

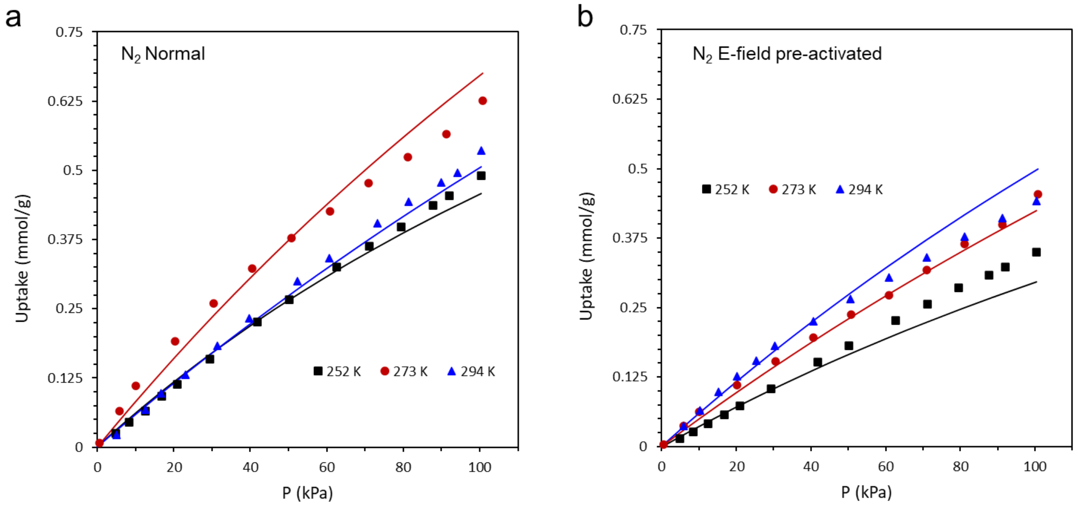

Supplementary Figure 7 Adsorption isotherms of N_2 in r2KCHA fitted with LJM-Toth model (a) without and (b) with the E-field pre-activation. Solid symbols refer to the experimental data with curves depicting the theoretical-fitting results using the LJM-Toth model.

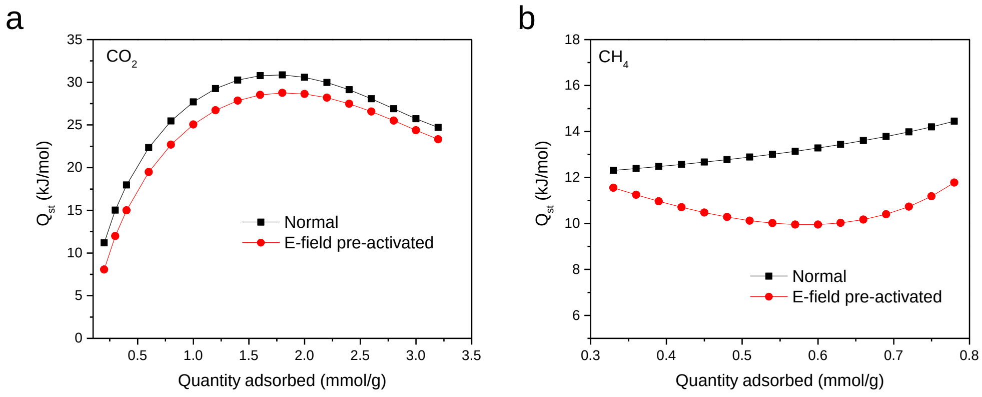

Supplementary Figure 8 The isosteric heat of adsorption Q_{st} of (a) CO_2 and (b) CH_4 in r2KCHA before and after the E-field pre-activation. The Q_{st} of CO_2 was calculated in the temperature range of 252 K to 294 K. The Q_{st} of CH_4 was calculated in the temperature range of 273 K to 323 K to avoid the influence of the trapdoor effect.

The heat of adsorption curve of CO_2 was first increased at the low coverage, which might be resulted from the strong interaction of the trapdoor cation and the CO_2 molecule at the beginning of adsorption. After a certain coverage, the increase of adsorbate–adsorbate interaction energy was limited by the adsorption capacity, while the drop of field gradient–quadrupole interaction, dispersion energy, repulsion energy, and polarization energy became dominant, finally leading to a decline in the Q_{st}.[1]

The heat of adsorption curve of CH_4 in a normal r2KCHA showed a slight increase with increasing coverage. However, after the E-field pre-activation, due to the reduced cation–molecule interaction caused by the relocation of partial K^+ in the pore, the Q_{st} of CH_4 in r2KCHA exhibited a declining trend at low coverages.

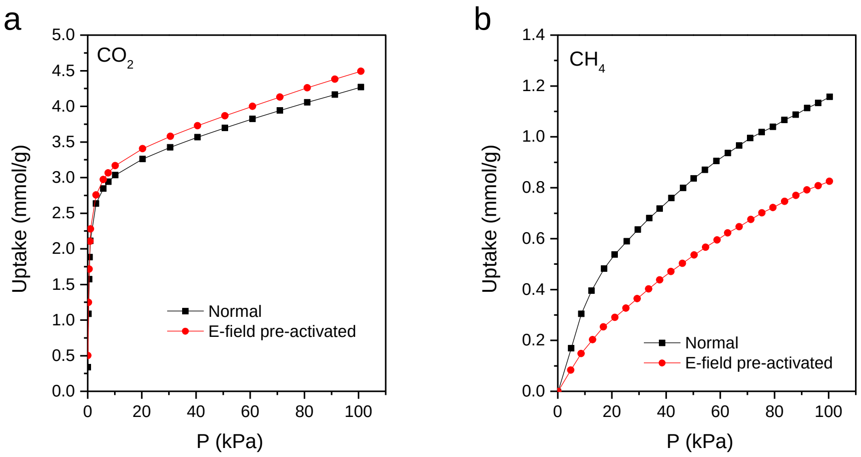

Supplementary Figure 9 Adsorption isotherms of (a) CO_2 and (b) CH_4 in r2KCHA at 273 K without and with the pre-activation of a direct current E-field at the intensity of 800 V/mm.

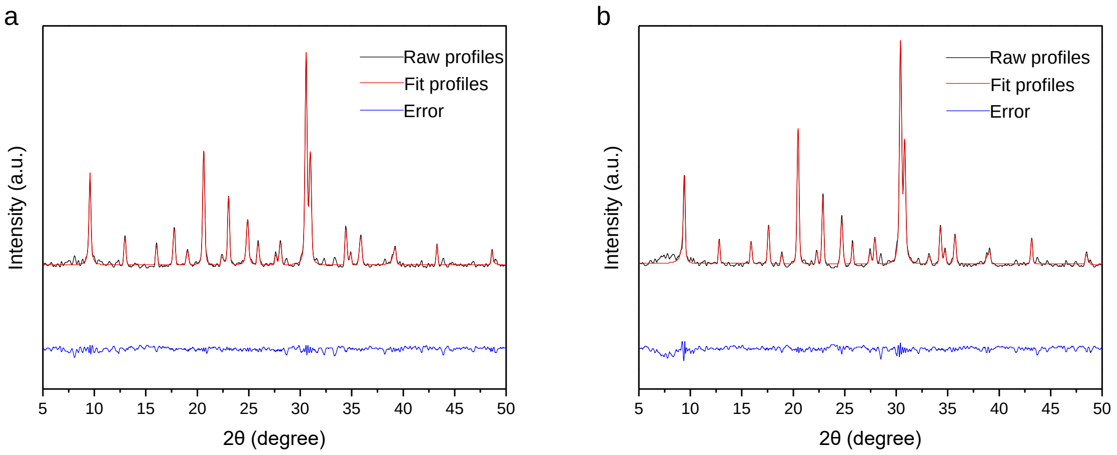

Supplementary Figure 10 PXRD patterns of r2KCHA and the fitted profiles calculated by MDI Jade 6.0. (a) The normal r2KCHA. (b) The E-field pre-activated r2KCHA.

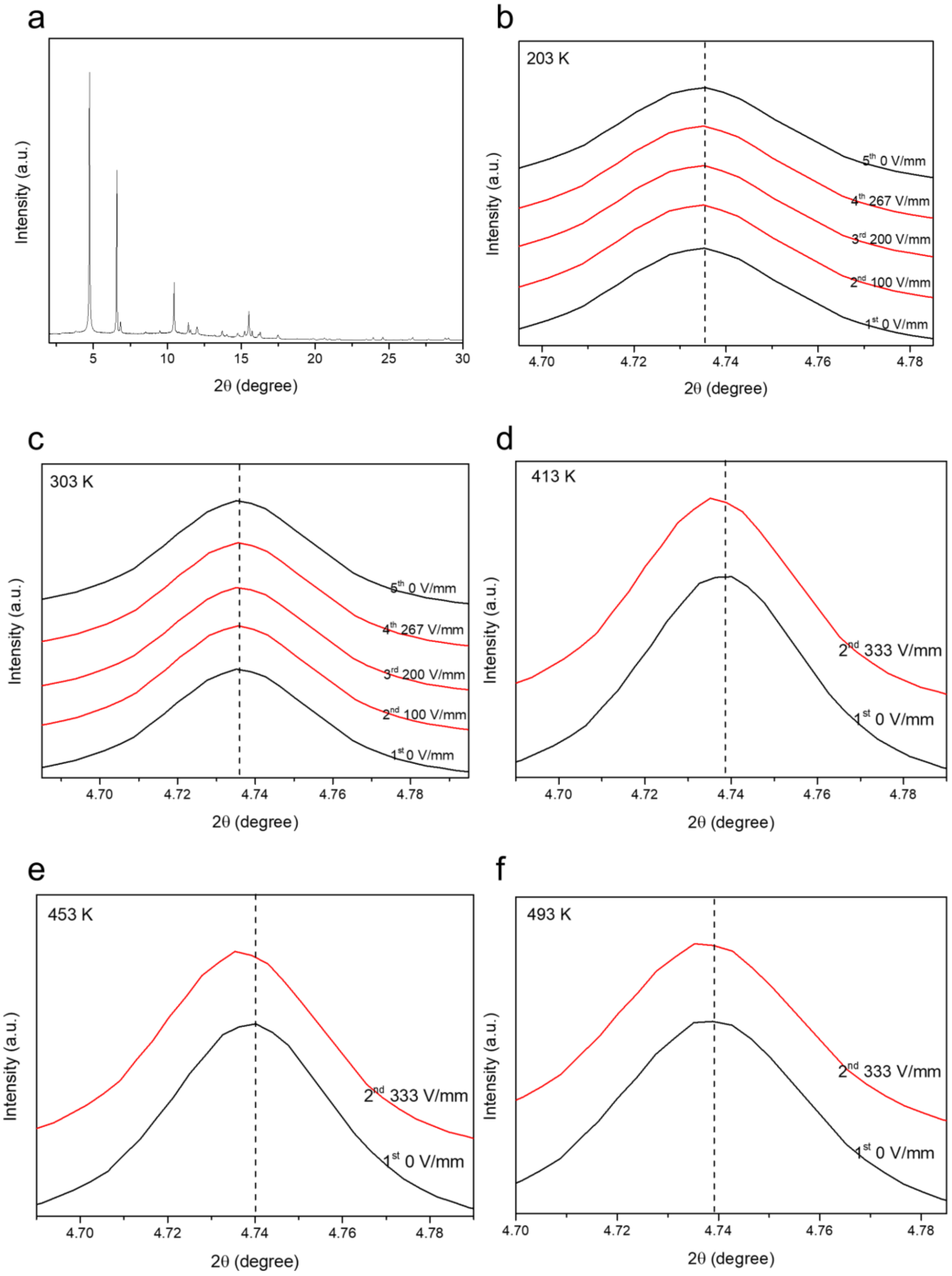

Supplementary Figure 11 Synchrotron PXRD measurements with the *in situ* E-field applications. (a) The complete PXRD pattern of r2KCHA. (b-f) PXRD patterns of r2KCHA with *in situ* E-field applications at (b) 203 K, (c) 303 K, (d) 413 K, (e) 453 K and (f) 493 K. The E-field intensity was 100, 200, 267 and 333 V/mm when the voltage was 120, 240, 320 V and

400 V respectively. At 203 and 303 K, the r2KCHA sample was successively exposed to the E-fields of 0, 100, 200, 267 and 0 V/mm. At 413, 453 and 493 K, the r2KCHA sample was successively exposed to the E-fields of 0 and 333 V/mm.

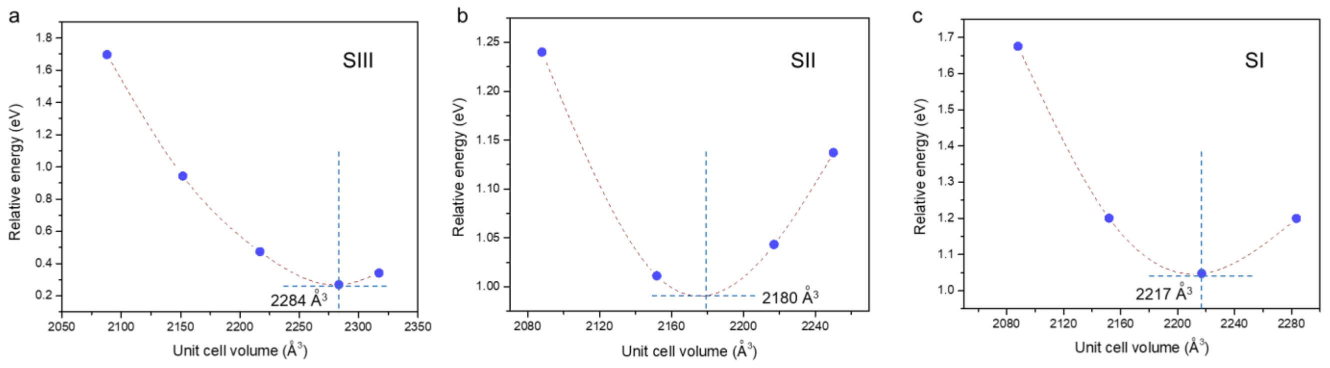

Supplementary Figure 12 Changes of relative energy over unit cell volume of r2KCHA with one K^+ moving from SIII' to (a) SIII, (b) SII and (c) SI.

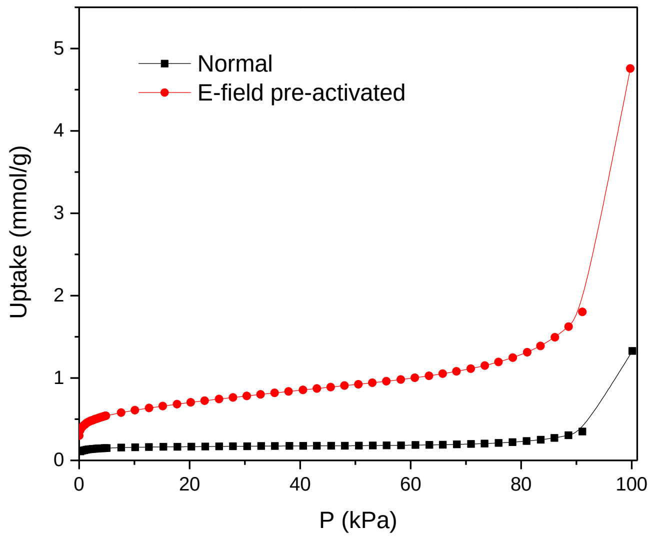

Supplementary Figure 13 Adsorption isotherms of N_2 in r2KCHA at 77 K without and with the E-field pre-activation.

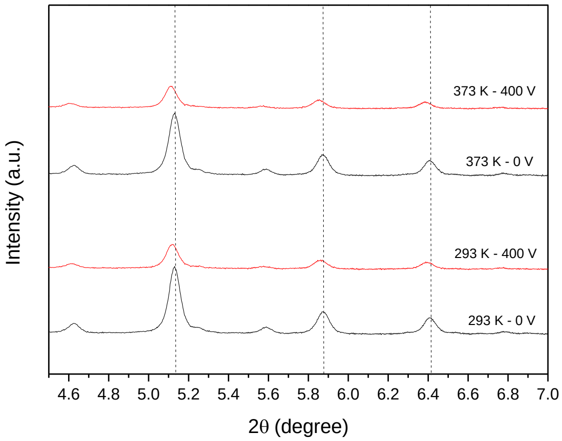

Supplementary Figure 14 Synchrotron PXRD analysis of ZSM-25-K under vacuum with *in situ* application of the E-field at 293 and 373 K. The E-field intensity was 200 V/mm when the voltage was 400 V. The X-ray wavelength was 0.6888 Å.

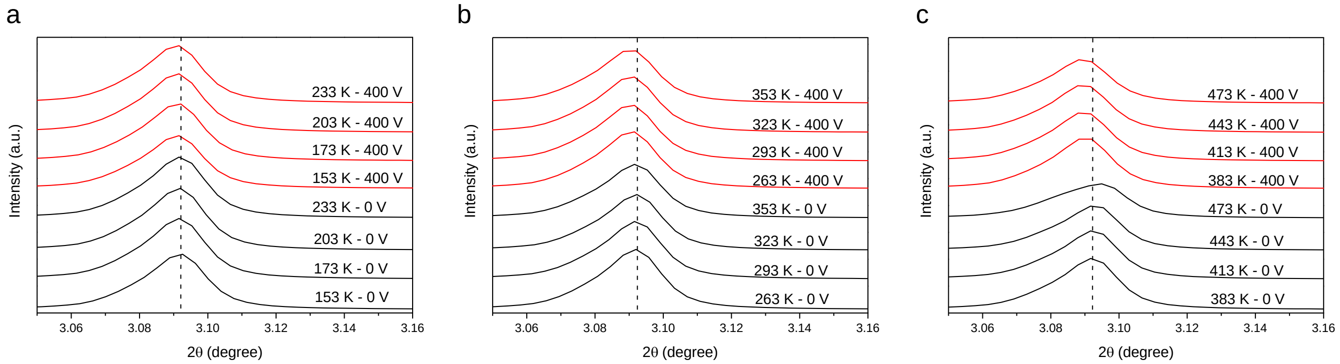

Supplementary Figure 15 Synchrotron PXRD analysis of TMA-Y under vacuum with *in situ* application of the E-field at (a) 153-233 K, (b) 263-353 K and (c) at 383-473 K. The E-field intensity was 333 V/mm when the voltage was 400 V. The X-ray wavelength was 0.7732 Å.

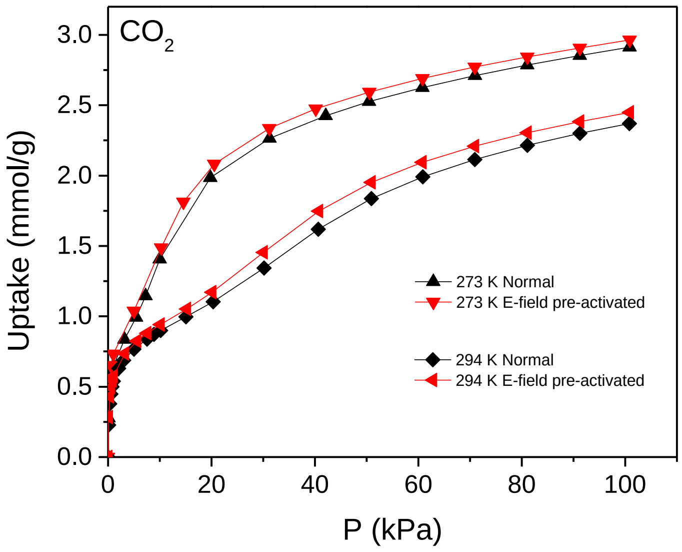

Supplementary Figure 16 Adsorption isotherms of CO_2 in ZSM-25-Na at 273 K and 294 K without and with the E-field pre-activation. The Na-ZSM-25 exhibited a rapid increase of CO_2 uptake at low pressures and a flat upward trend at high pressures, which was attributed to the framework expansion while adsorbing CO_2.[2]

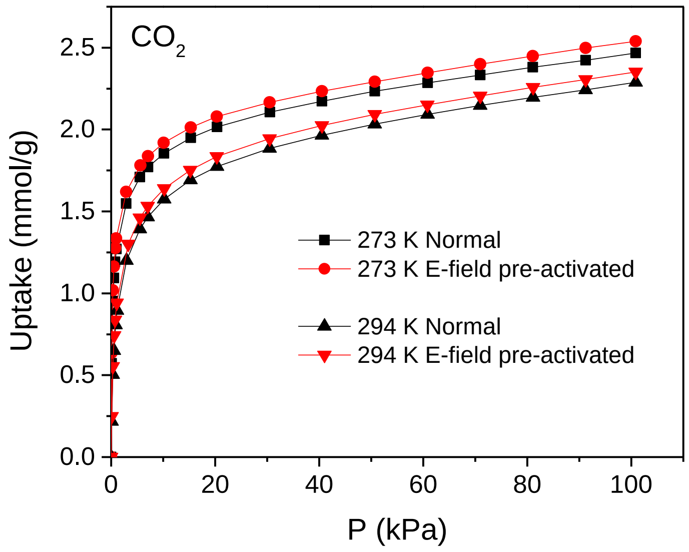

Supplementary Figure 17 Adsorption isotherms of CO_2 in ZSM-25-K at 273 K and 294 K without and with the E-field pre-activation.

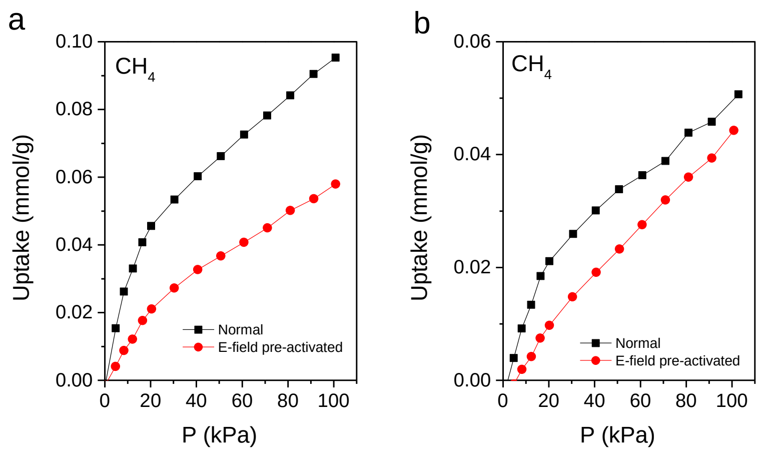

Supplementary Figure 18 Adsorption isotherms of CH_4 in ZSM-25-Na at (a) 273 K and (b) 294 K and without and with the E-field pre-activation.

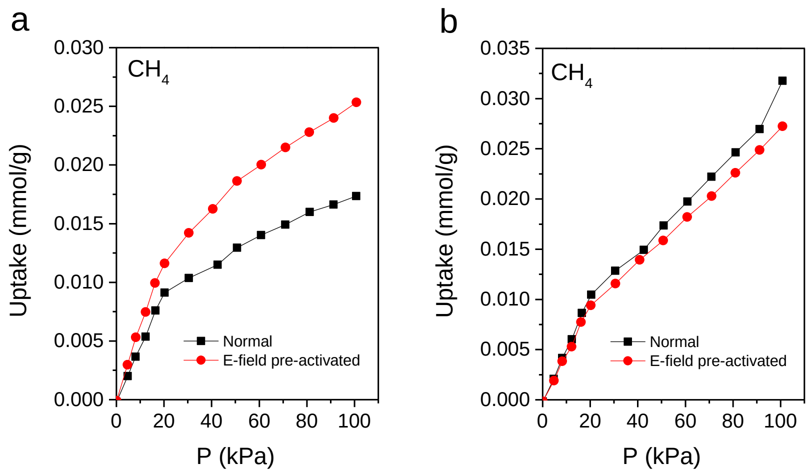

Supplementary Figure 19 Adsorption isotherms of CH_4 in ZSM-25-K at (a) 273 K and (b) 294 K without and with the E-field pre-activation.

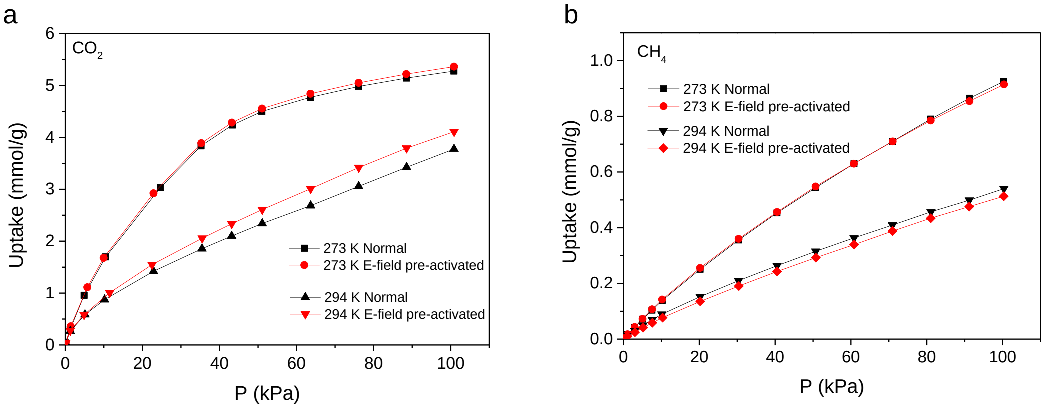

Supplementary Figure 20 Adsorption isotherms of (a) CO_2 and (b) CH_4 in TMA-Y without and with the E-field pre-activation.

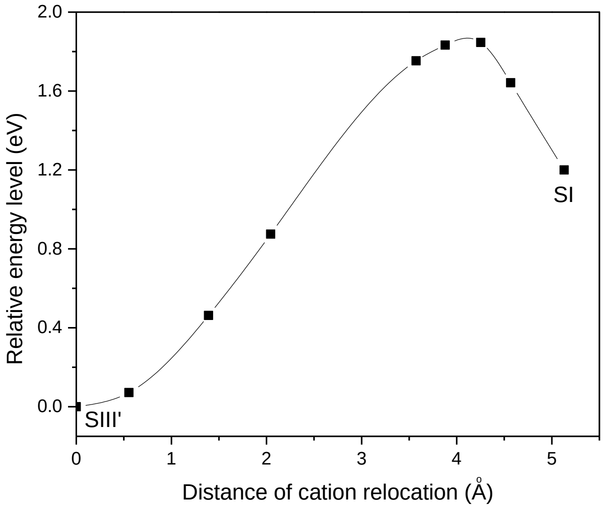

Supplementary Figure 21 The energy profile for one K^+ in r2KCHA relocating between SIII’ and SI calculated by DFT.

Supplementary Tables

Supplementary Table 1 Parameters of CO_2 adsorption isotherms of r2KCHA fitted with Toth model

	q_m (mol kg^{-1})	K^0	Q (kJ/mol)	t
Normal	4.34	2.05E-06	30.9	0.64
E-field pre-activated	4.53	9.76E-06	28.0	0.61

	T (K)	R^2
Normal	252	0.95
	273	0.96
	294	0.99
E-field pre-activated	252	0.95
	273	0.97
	294	0.99

Supplementary Table 2 Parameters of CH_4 adsorption isotherms of r2KCHA fitted with LJM-Toth model

	q_m (mol kg^{-1})	K^0	Q (kJ/mol)	t
Normal	1.62	3.90E-05	15.5	0.98
E-field pre-activated	1.35	1.05E-04	13.2	0.99

	T (K)	R^2
Normal	273	0.99
	294	0.99
	323	0.99
E-field pre-activated	273	0.99
	294	0.98
	323	0.99

Supplementary Table 3 Parameters of N_2 adsorption isotherms of r2KCHA fitted with LJM-Toth model

	q_m (mol kg^{-1})	K^0	Q (kJ/mol)	t
Normal	3.32	2.00E-05	11.0	0.99
E-field pre-activated	2.71	1.58E-04	6.02	0.99

	T (K)	R^2
Normal	252	0.99
	273	0.98
	294	0.99
E-field pre-activated	252	0.95
	273	0.93
	294	0.97

Supplementary Notes

Supplementary Note 1: The adsorption models used for the fit of adsorption isotherms of r2KCHA

The adsorption isotherms of CO_2 were fitted with the Toth model[3] as described in Equations (S1) and (S2),

$$q = q_m \frac{KP}{[1+(KP)^t]^{1/t}} \quad \text{(S1)}$$

$$K = K^0 \cdot \exp\left(-\frac{Q}{RT}\right) \quad \text{(S2)}$$

where q and q_m are the absolute adsorbed amount and the maximum adsorbed amount respectively (mol kg^{-1}), P is the pressure (kPa) for loading q, K^0 is the infinite adsorption constant, Q is the isosteric heat of adsorption at zero loading (kJ mol^{-1}), t is the heterogeneity parameter, T is the temperature of the system (K), and R is the gas constant (kJ kg^{-1} K^{-1}).

The adsorption isotherms of CH_4 and N_2 were fitted with the LJM-Toth model[4] in Equation (S3), which incorporated new parameters into classical adsorption models to describe the temperature-dependent pore accessibility of trapdoor materials.

$$q = [\Phi(1-\varepsilon) + \varepsilon] \frac{q_m KP}{[1+(KP)^t]^{1/t}} \quad \text{(S3)}$$

In Equation (S3), Φ is the temperature-dependent distribution function and ε is the fraction of external surface sites and defects always available for adsorption. The function $[\Phi(1-\varepsilon) + \varepsilon]$ determines the fraction of accessible adsorption sites at different temperatures, which is known as site accessibility. Based on the previous studies[4], the site accessibility of r2KCHA for CH_4 is 0.92 at 273 K. When the temperature is higher than 279 K, the trapdoor K^+ will be fully open and the LJM-Toth model will regress to the normal Toth model as the site accessibility is 1. For N_2, the site accessibility is 0.5 at 252 K and 1 at temperatures > 266 K. Other parameters are the same as those in the Toth model.

Supplementary Note 2: The heat of adsorption of CO_2, CH_4 and N_2 in r2KCHA

The isosteric heat of adsorption (Q_{st}) of CO_2 and CH_4 in r2KCHA was calculated by the Clapeyron equation in Equation (S4),

$$\frac{Q_{st}}{RT^2} = \left[\frac{\partial \mathrm{In} P}{\partial T}\right]_q \qquad \text{(S4)}$$

where Q_{st} (kJ mol^{-1}) is the isosteric heat of adsorption at the loading q (mol kg^{-1}), P is the pressure (kPa), T is the temperature of the system (K), and R is the gas constant (kJ kg^{-1} K^{-1}). If the isosteric heat is assumed to be independent of temperature, at a certain adsorption quantity, Equation (S4) can be integrated to give Equation (S5),

$$\ln P = \frac{Q_{st}}{RT} + C \qquad \text{(S5)}$$

where C is an integration constant. To calculate the Q_{st}, the isotherms at different temperatures were mathematically spline fitted to obtain ln(P) at a given adsorbed amount. The Q_{st} is then calculated by the slope of the linear equation of ln(P) versus $1/T$ in Equation (S5).

At the temperature range of 252 to 294 K, the N_2 adsorption in r2KCHA was strongly influenced by the trapdoor effect. The uptake was increased with the increasing temperature, disobeying the pattern of common physical adsorption. The experimentally measured N_2 uptake at these temperatures cannot reflect the real adsorption capacity due to the blocked pores. Therefore, its heat of adsorption cannot be calculated through the Clapeyron equation. The heat of adsorption of N_2 can be obtained from the fitted LJM-Toth model, as shown by the parameter Q in Supplementary Table 3.

Supplementary References:

1. Zhang J., Singh R. & Webley P. A. Alkali and alkaline-earth cation exchanged chabazite zeolites for adsorption based CO_2 capture. *Microporous Mesoporous Mater*. **111**, 478-487 (2008).

2. Zhao J., Xie K., Singh R., Xiao G., Gu Q., Zhao Q., Li G., Xiao P. & Webley P. A. Li^+/ZSM-25 zeolite as a CO_2 capture adsorbent with high selectivity and improved adsorption kinetics, showing CO_2-induced framework expansion. *J. Phys. Chem. C* **122**, 18933-18941 (2018).

3. Toth J. State equation of the solid-gas interface layers. *Acta chim. hung.* **69**, 311-328 (1971).

4. Li G. K., Shang J., Gu Q., Awati R. V., Jensen N., Grant A., Zhang X., Sholl D. S., Liu J. Z., Webley P. A. & May E. F. Temperature-regulated guest admission and release in microporous materials. *Nat. Commun.* **8**, 15777-15786 (2017).
